# A Decade Long Struggle: A Young Man’s Journey From Renal Transplant to Fatal Refractory Ascites

**DOI:** 10.7759/cureus.94290

**Published:** 2025-10-10

**Authors:** Sarah I Zahid, Nadia I Zahid, Mazaffar I Zahid, Imran Zahid

**Affiliations:** 1 Internal Medicine, Sheikh Shakhbout Medical City (SSMC), Abu Dhabi, ARE; 2 Family Medicine, Nassau University Medical Center, East Meadow, USA; 3 Internal Medicine, Gulf Medical Univeristy, Ajman, ARE; 4 Nephrology, Sheikh Shakhbout Medical City (SSMC), Abu Dhabu, ARE

**Keywords:** central venous occlusion, living donor renal transplant, lymphatic obstruction, refractory ascites, renal lymphangiectasia, transplant nephrectomy

## Abstract

This case details the clinical course of a 33-year-old man with a history of living with an unrelated renal transplant, who presented with recurrent, massive ascites to be drained every two to three weeks for two years. His post-transplant course was also complicated by chronic antibody-mediated rejection, evidenced by a terminal increase in serum creatinine and a decrease in estimated glomerular filtration rate. Cross-sectional imaging revealed complete obliteration of his central veins and a hypertrophied, edematous transplant kidney anastomosed to the common iliac artery. These findings, in the absence of cirrhosis or cardiac failure, pointed towards renal lymphangiectasia as a consequence of venous outflow obstruction and possible iatrogenic lymphatic injury as the cause of his refractory ascites. This case reinforces the critical role of venous and lymphatic pathology in the differential diagnosis of post-transplant ascites and demonstrates how a multifactorial interaction can lead to a disastrous clinical scenario.

## Introduction

A kidney transplant represents a return to health and independence from dialysis for thousands of patients [1]. However, this gift of life requires lifelong careful management to navigate complications, which can range from rejection and infection to technical surgical issues [2]. Among the more challenging surgical complications are injuries to the lymphatic network surrounding the transplant site. Typically causing manageable fluid collections known as lymphoceles, the injuries can, in severe cases, lead to a debilitating and relentless accumulation of fluid in the abdomen, known as refractory ascites [3, 4].

The ascites after a transplant can have many causes, but when linked directly to the new kidney, a rare condition called renal lymphangiectasia must be considered [5]. This occurs when the lymphatic vessels within the kidney become dilated and leaky, often because the veins draining the kidney are obstructed, leading to a backup of pressure [6, 7]. Diagnosing this condition requires a high degree of suspicion [8].

We present the case of a young man who battled for nearly a decade following his transplant. His case serves as a reminder of how technical challenges during surgery, the body's immune response, and unforeseen vascular events can intertwine, leading to a complex and ultimately tragic outcome [9, 10]. Our goal is to honor his difficult journey by highlighting the lessons it offers to the medical community.

## Case presentation

In 2014, a 33-year-old man, after years of dialysis and kidney failure, received a living donor left kidney transplant abroad. The surgery was lengthy and complex, as doctors navigated through layers of scar tissue from his past medical battles. The new kidney was connected to a major artery in his pelvis, the common iliac artery.

**Figure 1:**
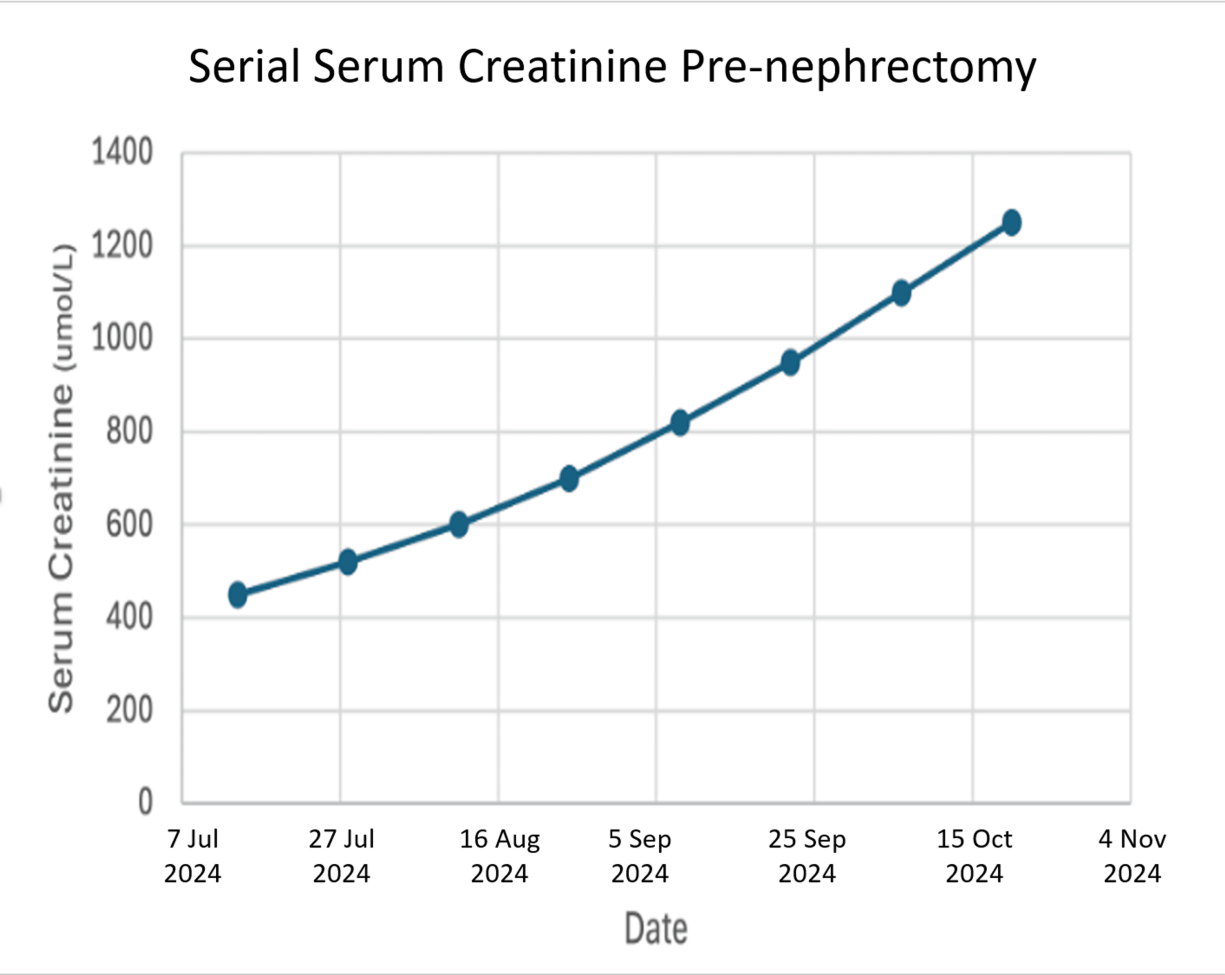
Serial serum creatinine levels pre-nephrectomy

**Figure 2:**
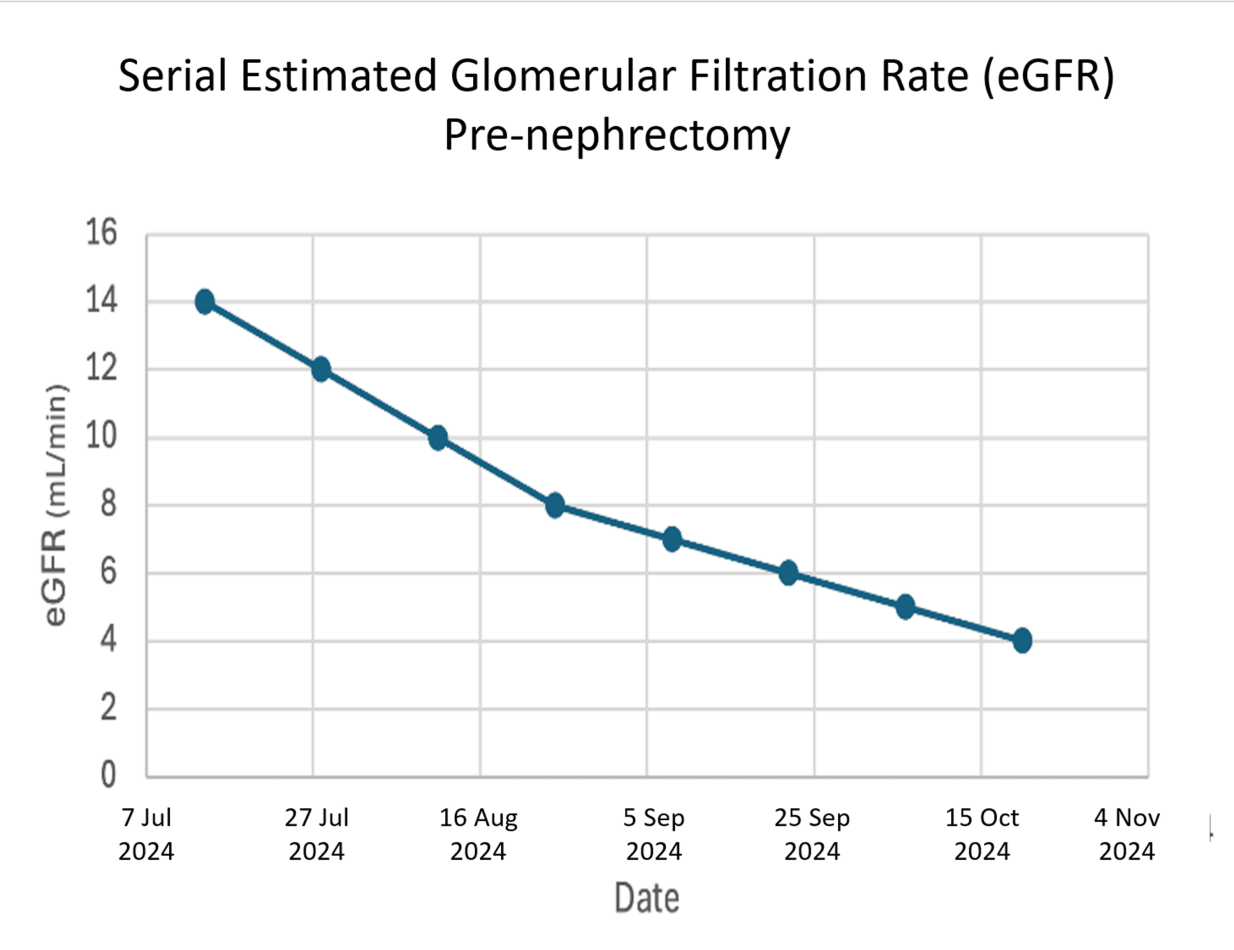
Serial estimated glomerular filtration rate (eGFR) pre-nephrectomy

The early years following the transplant were hopeful, but issues later arose. He battled a viral infection of the kidney (BK virus). He later developed chronic rejection of the organ, identified on kidney graft biopsy, a common but serious long-term threat to transplant survival [11, 12]. He remained on a course of anti-rejection medications (tacrolimus, mycophenolate mofetil, and prednisone) to preserve his kidney function. Serial laboratory follow-up over the months prior to the removal of the transplant graphically illustrated its terminal failure, as reflected by a rising serum creatinine (Figure 1) and a corresponding fall in estimated glomerular filtration rate (Figure 2), which confirmed end-stage graft failure.

Then, around 2019, a new problem arose: an enormous, recurrent accumulation of fluid in his abdomen. This ascites was so severe that he had to be admitted to the hospital every few weeks for drainage, dramatically impacting his quality of life. Investigations revealed the shocking extent of his condition. CT scans showed that his inferior vena cava and other central veins were occluded, with his body having formed a network of collateral veins to bypass the obstructions. His transplanted kidney was swollen and congested.

**Figure 3:**
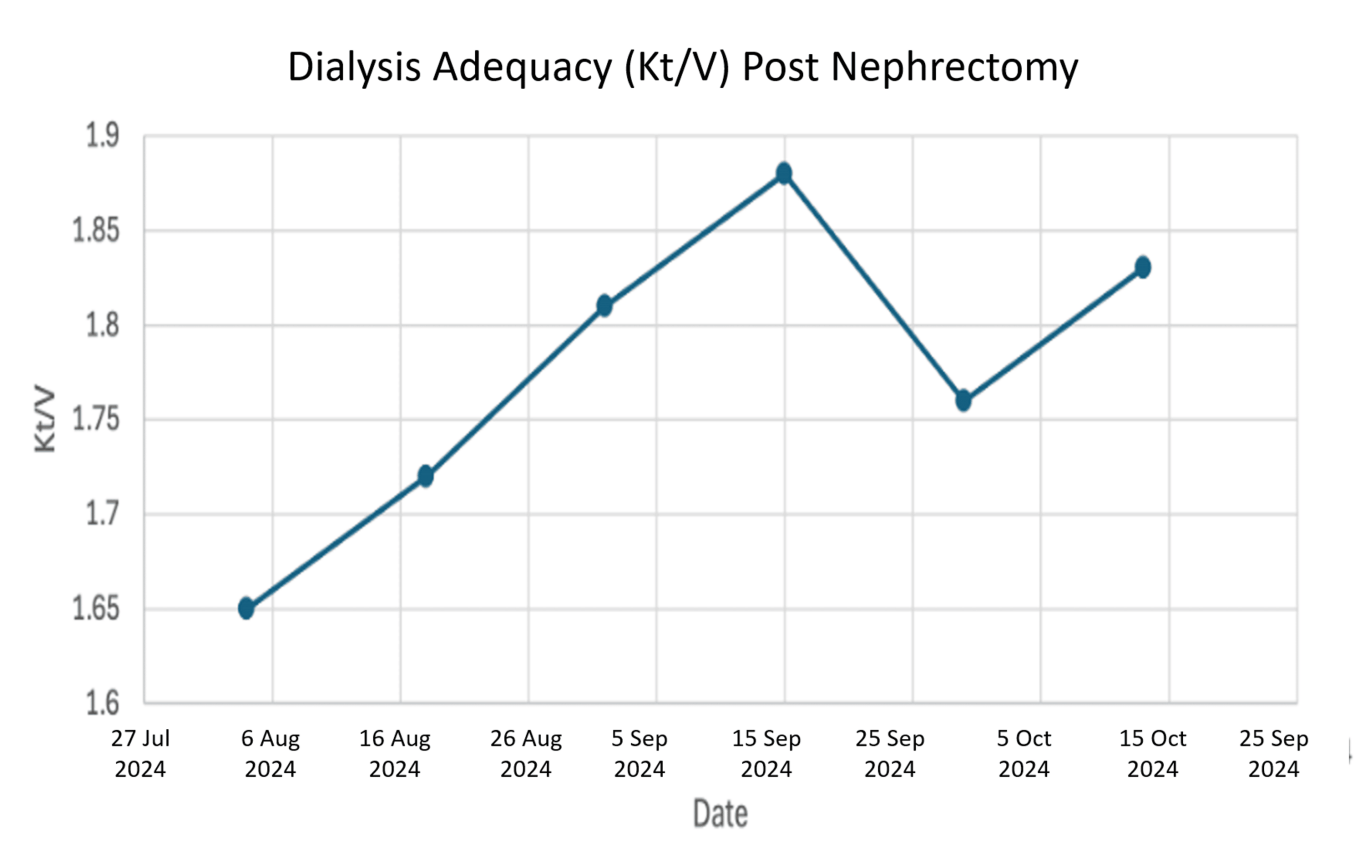
Dialysis adequacy (Kt/V) post nephrectomy

**Figure 4:**
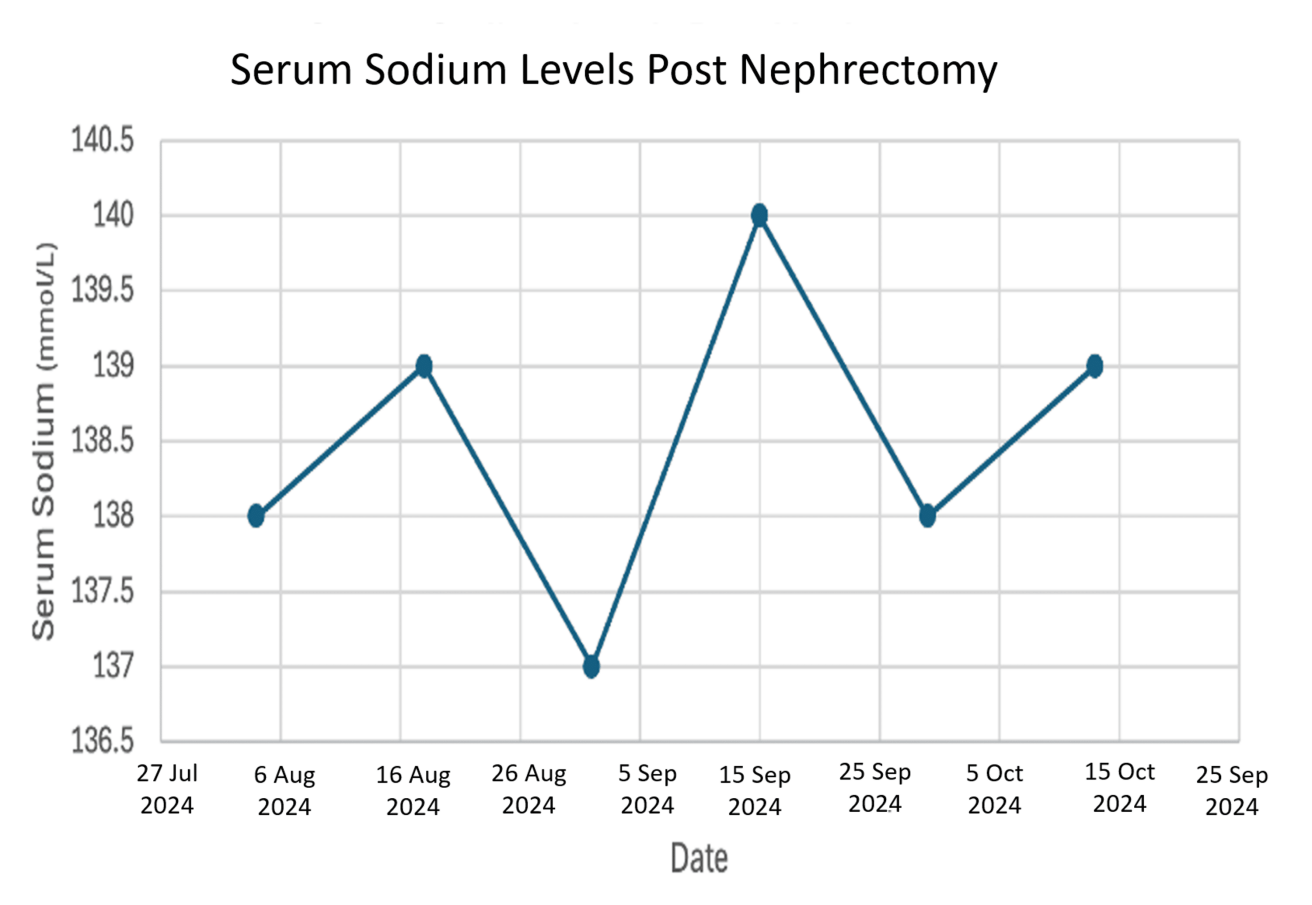
Serum sodium levels post nephrectomy

After ruling out other common causes like liver disease, his physicians concluded that the widespread vein blockages had created a backup of pressure, causing the kidney's lymphatic system to rupture and leak fluid, a condition known as renal lymphangiectasia [6, 7]. With limited options to reverse the vein blockages and his transplanted kidney failing, the difficult decision was made to remove the left kidney transplant in 2023, returning him to dialysis. Post-nephrectomy, he was maintained on regular hemodialysis post nephrectomy, with Kt/V measurements confirming adequate dialysis clearance (Figure 3) and stable serum sodium levels (Figure 4).

His final years were also marked by a personal battle with chronic pain and the complexities of pain management. He was briefly hospitalized in October 2024 to address a malfunctioning dialysis catheter and was discharged in stable condition. He passed away the following day.

## Discussion

This patient's trajectory represents a devastating amalgamation of multiple medical complications. His refractory ascites was not due to a single cause but rather the result of three interconnected factors that cumulatively created a perfect storm.

The diagnosis of this complex condition was pieced together through a multi-modal approach. The cornerstone for assessing the graft was the use of serial kidney biopsies, which definitively identified the key pathologies: a biopsy in 2014 confirmed BK virus nephropathy, and a later biopsy in 2021 provided histologic evidence of chronic antibody-mediated rejection and interstitial fibrosis. The etiology of the ascites and the overarching vascular compromise was elucidated through CT angiography. These studies objectively revealed the extensive central venous occlusions and the swollen, edematous state of the transplant kidney, which, after ruling out hepatic causes, led to the diagnosis of renal lymphangiectasia as the mechanism for the recurrent ascites.

First, the initial transplant procedure was technically demanding. Operating in a scarred field increases the danger of damage to the network of lymphatic channels that run alongside the blood vessels [4, 10]. While surgeons take great care to ligate these vessels, a difficult dissection can sometimes lead to persistent leakage, which in this patient may have established the groundwork for his later troubles [13, 14].

Second, his body began to chronically reject the kidney. This rejection process is not just an immune attack but also causes inflammation and scarring within the organ itself [15, 16]. This internal scarring would have further impaired the kidney's delicate drainage systems, including its lymphatic vessels, making it even more vulnerable to pressure changes [17, 18]. The laboratory data (Figures 1, 2) provide objective evidence of this progressive functional decline.

The third and most significant factor was the development of extensive blockages in his central veins. Such blockages are a known risk for patients with a history of multiple dialysis catheters and can lead to severe venous hypertension [19, 20]. For a transplanted kidney, which relies on patent veins for drainage, this is catastrophic. The obstructed outflow created enormous pressure within the kidney, forcing a large volume of fluid into its lymphatic system, a system likely already compromised by the initial surgery and chronic rejection. The pressure eventually led to the rupture of the lymphatic vessels, leading to the relentless ascites that marked the last few years of his life [6]. His swollen kidney on imaging was a characteristic sign of this pressure overload, and the extensive collateral veins were his body's attempt to provide a bypass for blood to return to his heart [20].

The question of potential interventions for this condition is critical. For renal lymphangiectasia, options like percutaneous drainage or surgical fenestration can be considered, while venous stenting may relieve hypertension in cases of focal venous obstruction [19,20]. However, the diffuse and chronic nature of his central venous occlusions, combined with the transplant kidney's end-stage failure from irreversible rejection, rendered him a poor candidate for these approaches. The extensive collateral circulation seen on imaging underscored the chronicity and irreversibility of the process. Consequently, management was necessarily palliative, focused on recurrent paracentesis for symptomatic relief.

We can now construct a likely timeline: the surgical challenge may have caused an initial lymphatic injury. The chronic rejection then caused progressive scarring within the kidney. Finally, the central vein occlusions acted as the tipping point, creating an overwhelming pressure problem that culminated in the devastating clinical picture of refractory ascites. The adequacy of his subsequent dialysis (Figure 3) and stable electrolytes (Figure 4) confirms that his ultimate death was not due to inadequate renal replacement therapy or metabolic derangement, but rather the cumulative burden of these complex, interrelated pathologies.

This case emphasizes that, in the context of post-transplant ascites, more than the usual etiologies must be considered by clinicians. A high index of suspicion for venous and lymphatic complications is essential [5, 8]. Furthermore, a structured diagnostic workup incorporating graft biopsy for intrinsic pathology and CT angiography for vascular anatomy is critical for identifying the multifaceted causes seen in such complex presentations. While the extent of blockages in this case may have been untreatable, earlier recognition of this mechanism might have prompted a different discussion regarding potential interventions.

## Conclusions

This is not just a medical case but the story of a patient who had to endure a decade-long struggle after his transplant. His experience highlights the reality that even life-saving interventions can have rare and cascading effects. His refractory ascites was the final manifestation of a complex interplay between a difficult surgery, chronic rejection, and catastrophic venous disease, with objective data tracing the graft’s failure and his dialysis course afterward. This case emphasizes the profound challenges some transplant recipients face and the dire need for a proactive, multidisciplinary approach to manage such complex situations. By publicizing this case, we hope to heighten awareness and encourage earlier treatment for future patients who develop signs of this devastating complication cascade.

## References

[1] Hart A, Lentine KL, Smith JM (2021). OPTN/SRTR 2019 annual data report: kidney. Am J Transplant.

[2] Divakar D, Bailey RR, Lynn KL, Robson RA (1991). Long term complications following renal transplantation. N Z Med J.

[3] Ranghino A, Segoloni GP, Lasaponara F, Biancone L (2015). Lymphatic disorders after renal transplantation: new insights for an old complication. Clin Kidney J.

[4] Atray NK, Moore F, Zaman F, Caldito G, Abreo K, Maley W, Zibari GB (2004). Post transplant lymphocele: a single centre experience. Clin Transplant.

[5] Lerut T, Lerut J, Broos P, Gruwez JA, Michielsen P (1980). Lymphatic complications in renal transplantation. Eur Urol.

[6] Hamroun A, Puech P, Maanaoui M, Bouyé S, Hazzan M, Lionet A (2021). Renal lymphangiectasia, a rare complication after kidney transplantation. Kidney Int Rep.

[7] Baker ML, Cantley LG (2023). The lymphatic system in kidney disease. Kidney360.

[8] Lu X, Ma K, Ren J (2024). The immune regulatory role of lymphangiogenesis in kidney disease. J Transl Med.

[9] Humar A, Matas AJ (2005). Surgical complications after kidney transplantation. Semin Dial.

[10] Yamanaka K, Kakuta Y, Nakazawa S, Kobayashi K, Nonomura N, Kageyama S (2025). Surgical and infectious complications following kidney transplantation: a contemporary review. J Clin Med.

[11] Hirsch HH, Randhawa PS (2019). BK polyomavirus in solid organ transplantation-guidelines from the American Society of Transplantation infectious diseases community of practice. Clin Transplant.

[12] Puttarajappa C, Shapiro R, Tan HP (2012). Antibody-mediated rejection in kidney transplantation: a review. J Transplant.

[13] Chin Ai, Ragavendra N, Hilborne L, Gritsch HA (2003). Fibrin sealant sclerotherapy for treatment of lymphoceles following renal transplantation. J Urol.

[14] Mohamed IH, Bagul A, Doughman T, Nicholson ML (2012). Use of internal iliac artery as a side-to-end anastomosis in renal transplantation. Ann R Coll Surg Engl.

[15] Nankivell BJ, Chapman JR (2006). Chronic allograft nephropathy: current concepts and future directions. Transplantation.

[16] Fadili W, Habib Allah M, Laouad I (2013). Chronic renal allograft dysfunction: risk factors, immunology and prevention. Arab J Nephrol Transplant.

[17] Donnan MD, Kenig-Kozlovsky Y, Quaggin SE (2021). The lymphatics in kidney health and disease. Nat Rev Nephrol.

[18] Liu WC, Kuo MC, Wu WJ, Hwang SJ, Chen HC (2006). Chylous ascites after renal transplantation-a case report. Nephrol Dial Transplant.

[19] Lumsden AB, MacDonald MJ, Isiklar H, Martin LG, Kikeri D, Harker LA, Allen RC (1997). Central venous stenosis in the hemodialysis patient: incidence and efficacy of endovascular treatment. Cardiovasc Surg.

[20] Shi YX, Ye M, Liang W, Zhang H, Zhao YP, Zhang JW (2013). Endovascular treatment of central venous stenosis and obstruction in hemodialysis patients. Chin Med J (Engl).

